# A time-series analysis of the short-term effects of daily PM_2.5_ exposure on cause-specific mortality in Mashhad, Iran (2019–2024)

**DOI:** 10.1038/s41598-026-48267-y

**Published:** 2026-04-11

**Authors:** Majid Kermani, Ehsan Mosa Farkhani, Fatemeh Joulaei, Ahmad Jonidi Jafari, Abbas Shahsavani

**Affiliations:** 1 Research Center for Environmental Health Technology, Iran University of Medical Sciences, Tehran, Iran; 2 Department of Environmental Health Engineering, School of Public Health, Iran University ofMedical Sciences, Tehran, Iran; 3 Department of Epidemiology and Biostatistics, School of Health, Mashhad University of MedicalSciences, Khorasan Razavi, Iran; 4 Department of Environmental Health Engineering, School of Public Health and Safety, Shahid Beheshti University of Medical Sciences, Tehran, Iran

**Keywords:** PM_2.5_, Mortality, Time series, Quasi-Poisson GAMs model, Air pollution, Diseases, Environmental sciences, Medical research, Risk factors

## Abstract

Fine particulate matter (PM_2.5_) is a major environmental risk factor associated with increased mortality from cardiovascular and respiratory diseases worldwide. This study examined the short-term association between daily PM_2.5_ exposure and cause-specific mortality in Mashhad, a large metropolitan city in northeastern Iran, over the period from March 2019 to March 2024. Daily concentrations of PM_2.5_ were obtained from a city-wide air quality monitoring network, and mortality data for ischemic heart disease, stroke, chronic obstructive pulmonary disease, acute lower respiratory infection, asthma, and lung cancer were collected from official health records. A time-series analysis was conducted using generalized additive models with a quasi-Poisson distribution to estimate relative risks and 95% confidence intervals associated with a 10 µg/m³ increase in PM_2.5_ concentrations. Both single-day lag effects (0–5 days) and cumulative lag effects were evaluated, while accounting for temporal trends and meteorological variables. The results showed that short-term increases in PM_2.5_ concentrations were significantly associated with higher mortality from ischemic heart disease, stroke, chronic obstructive pulmonary disease, and acute lower respiratory infection, with the strongest effects generally observed at lag days 4–5 and cumulative lag periods. No statistically significant short-term associations were detected for asthma or lung cancer mortality. In addition, PM_2.5_ concentrations consistently exceeded the World Health Organization guideline during most of the study period, indicating sustained population exposure to elevated air pollution levels. These findings provide evidence that short-term exposure to PM_2.5_ contributes to increased cause-specific mortality in an urban Middle Eastern setting and highlight the public health importance of effective air pollution control strategies to reduce preventable deaths.

## Introduction

Non-communicable diseases (NCDs), particularly cardiovascular diseases, strokes, and chronic respiratory diseases, are recognized as one of the most significant global health challenges, accounting for the largest share of mortality and disability each year. According to the World Health Organization (WHO), approximately 74% of global deaths are attributed to NCDs, among which ischemic heart disease and stroke are the leading causes of mortality^1^. In Iran, similar to many developing countries, ischemic heart diseases have been reported as the primary cause of death, and the disability-adjusted life years (DALY) associated with these diseases are considerably higher compared to other conditions^2^.

Among the individual and environmental risk factors influencing these diseases, air pollution is recognized as one of the most important environmental risk factors. According to WHO estimates, approximately 7 million premature deaths worldwide are attributed to air pollution annually, with more than 6% of total mortality related to particulate matter exposure^2^. Extensive studies indicate that exposure to air pollutants is associated with the incidence of a wide range of diseases and increased mortality. Major health outcomes of such exposure include IHD, LC, COPD, ALRI, stroke, and short-term hospitalizations due to cardiovascular and respiratory conditions. These conditions not only impose a substantial burden on public health but also result in significant economic and social consequences. According to the WHO, nearly 80% of deaths from IHD and stroke, 14% of deaths related to COPD and ALRI, and 6% of deaths due to LC are attributable to ambient air pollution^3^.

Air pollution refers to a mixture of suspended particles, gases, and chemical and biological compounds in the atmosphere that can threaten human health and the environment. Among air pollutants, particulate matter with a diameter of less than 2.5 microns (PM_2.5_) is considered the most hazardous component due to its specific characteristics, including extremely small size, the ability to penetrate deep into the respiratory tract and enter the bloodstream, as well as its capacity to carry toxic chemical compounds. PM_2.5_ originates from various sources such as exhaust emissions^4,5^, biomass burning, coal combustion, industrial emissions^6,7^, dust^4,7^, and secondary inorganic aerosols (formed from atmospheric reactions of NOₓ, SO_2_, and NH_3_, accounting for 21–48% of PM_2.5_ mass)^7^. Numerous studies conducted in different countries have shown that an increase in daily concentrations of PM_2.5_ is directly associated with higher mortality from cardiovascular diseases, strokes, and respiratory illnesses^8^. Furthermore, epidemiological evidence indicates that short-term exposure to high levels of PM_2.5_ can contribute to increased daily mortality as well as higher hospital admissions due to cardiovascular and respiratory diseases. Time-series and case-crossover studies in developed countries have repeatedly confirmed this association. In a study by Amoatey et al., it was found that long-term exposure to elevated PM_2.5_ concentrations was associated with increased mortality rates of 27.67%, 15.90%, 9.50%, and 19.90% for IHD, COPD, LC, and stroke, respectively^9^. Zhang et al. evaluated the relationship between non-communicable diseases and PM_2.5_ concentrations in 2017, reporting 130.22 and 204.07 deaths attributable to PM_2.5_ exposure from IHD and cardiovascular diseases (CD), respectively^10^. Oh et al. reported that, out of a total of 2,749,704 deaths in Korea in 2019, approximately 1,039 deaths were linked to short-term exposure to PM_2.5_^11^. A meta-analysis and cohort study further demonstrated that long-term exposure to elevated PM_2.5_ concentrations could increase the risk of mortality from COPD and IHD by 2.5% and 10%, respectively^12,13^. In addition to these international studies, domestic research, including the CAPACITY study in Isfahan^2^, the assessment of particulate effects in Ahvaz^14^, and evaluations of ill-health outcomes using the AirQ+ software^3^, has also reported a significant association between air pollution and hospitalizations or deaths due to cardiovascular and respiratory diseases. Nevertheless, local scientific evidence on the association between daily variations in PM_2.5_ concentrations and mortality from NCDs in Iranian cities remains limited.

Mashhad, as one of the major metropolitan cities of Iran and a leading destination for religious and cultural tourism, has been experiencing population growth and industrial development^15,16^. These factors have recently contributed to increased emissions of pollutants and a decline in air quality, such that available data indicate that the average concentration of air pollutants particularly PM_2.5_ has been rising in Mashhad and, in some seasons, especially winter, has exceeded the WHO standards^17^. Considering the upward trend of air pollution in this metropolitan city of Iran, along with the significant role of NCDs in the mortality pattern, conducting precise investigations in this field is essential. Accordingly, the present study was conducted to examine the association between daily variations in PM_2.5_ concentrations and daily mortality attributable to IHD, stroke, COPD, ALRI, asthma and LC in Mashhad during 21 March 2019 to 19 March 2024. In this study, generalized ensemble models (GAM) based on time series with a quasi-Poisson distribution were used to investigate the effect of PM_2.5_ concentration on daily hospitalizations and explore the exposure-lag-day-response associations.

## Materials and methods

### Study area

**Fig. 1:**
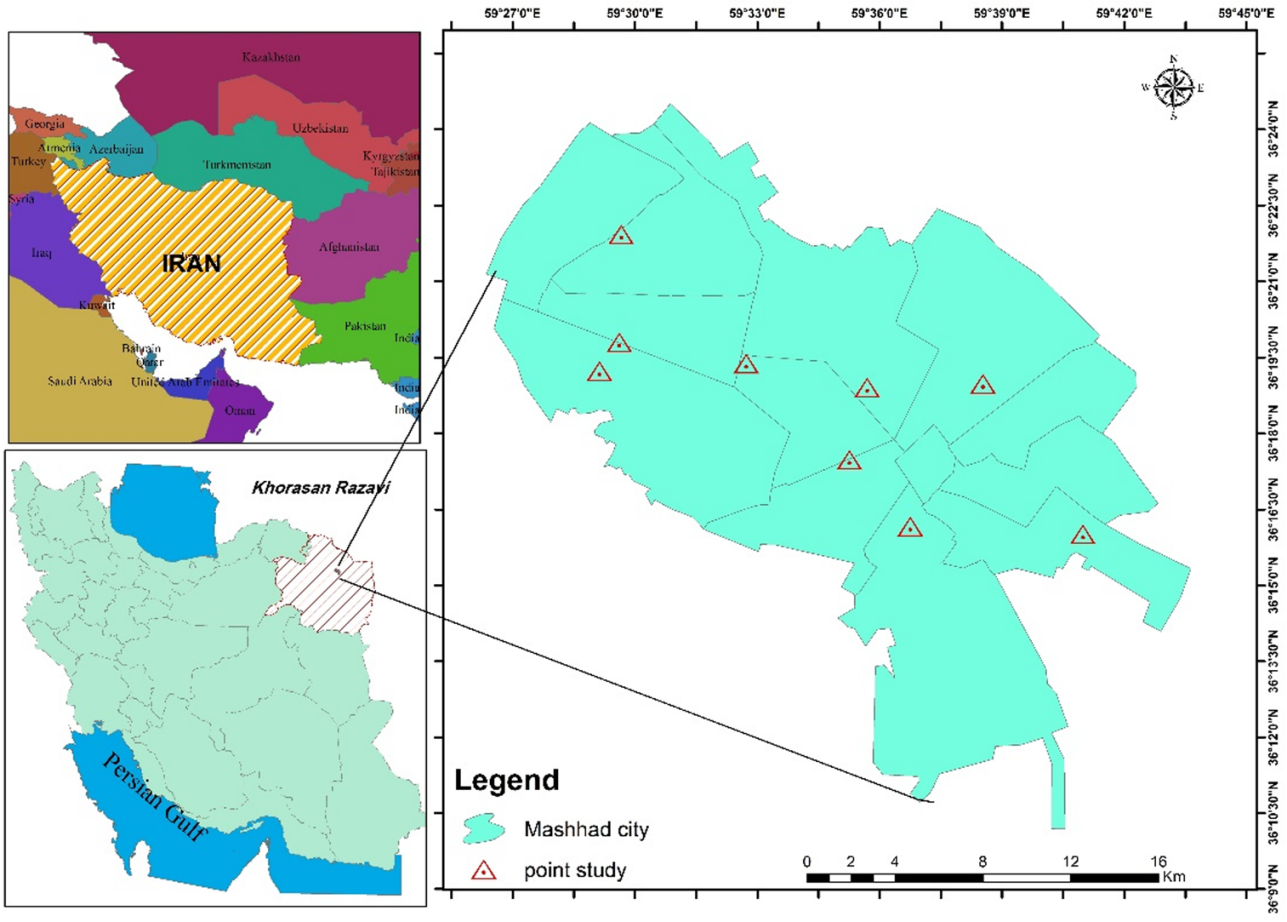
Location of the study area and air quality monitoring stations.

Mashhad, the capital of Razavi Khorasan Province, is located on the southern edge of the Kashafrud River at the geographical coordinates of 36°16′ N latitude and 59°38′ E longitude. With an area of 351 km^2^ and a population of 3,001,184, it is recognized as the second-largest city in Iran after Tehran^18^. Men and women constitute 49.9% and 50.1% of the population, respectively. The population distribution of Mashhad consists of 24.2% under 15 years, 58.2% aged 15–64 years, and 27.6% over 64 years^16^. The city of Mashhad has 23 air quality monitoring stations that continuously measure key air indicators, including particulate matter, ozone, sulfur dioxide, nitrogen dioxide, and carbon monoxide. Figure 1 illustrates the geographical location and coordinates of Mashhad.

### Ambient air pollutants and meteorological data

In this study, daily PM_2.5_ air quality data were collected from 23 monitoring stations in Mashhad during the period from 21 March 2019 to 19 March 2024. Due to various operational and calibration issues associated with air quality monitoring stations, a considerable portion of the data often lacks sufficient validity for use in research and analysis. Therefore, data processing of the monitoring station outputs was necessary and carried out in the present study as follows. First, the data from all monitoring stations were compiled, and the number of measurement hours for each pollutant at each station during the study years was determined. At this stage, all zero and negative PM_2.5_ values were removed. Hourly PM_2.5_ data were screened for outliers using a Z-score approach, which is commonly applied in air quality time-series analyses. Observations with absolute Z-scores greater than 4 were considered outliers and excluded from further analysis, following established practices for quality control of environmental monitoring data^18^.

Next, the compiled PM_2.5_ data were subjected to a structured quality control process. First, zero and negative values were removed. Hourly PM_2.5_ observations were then screened for outliers using a Z-score approach, with values exceeding an absolute Z-score of 4 excluded from the analysis. After data cleaning, only monitoring stations with at least 50% valid hourly measurements were retained. Finally, validated hourly data were aggregated to calculate 24-hour mean PM_2.5_ concentrations for subsequent analyses.In this study, Z-scores were calculated for hourly data to identify outliers. Data points with Z-scores outside the range of ± 4 (i.e., greater than + 4 or less than − 4) were considered outliers and excluded from the analysis. This threshold was selected based on the assumption of a normal distribution, in which more than 99% of the data are expected to fall within ± 4 standard deviations from the mean^19^. Less than 2% of our data were identified as outliers. After data preprocessing, only stations with at least 50% of hourly measurements were included in the study^20^. Fortunately, a preliminary assessment showed that the proportion of outliers in several stations was so small that, after preprocessing, all of them met the validity criteria and were included as eligible stations. After identifying reliable stations, data coverage was assessed to evaluate changes in descriptive statistics before and after processing. If only minor and insignificant variations were observed in the processed data, the processing was repeated. Finally, 24-h and annual average concentrations of PM_2.5_ were calculated from the validated hourly data^18,21^. The meteorological data required for this study, including daily mean temperature, relative humidity, and air pressure, were obtained from the Iranian Meteorological Organization and the NOAA website (https://www.noaa.gov).

### Mortality data

Non-accidental mortality data related to IHD, stroke, COPD, ALRI, asthma and LC from 21 March 2019 to 19 March 2024 were obtained from the database of the Iranian Ministry of Health and Medical Education. Causes of death were coded according to the International Classification of Diseases, 10th Revision (ICD-10), and mortality data were categorized into respiratory diseases (ICD-10: J00–J99), cardiovascular diseases (ICD-10: I00–I99), IHD (ICD-10: I20–I25), and cerebrovascular diseases (ICD-10: I60–I69).

### Ethical approval and consent

The study protocol was reviewed and approved by the Ethics Committee of Iran University of Medical Sciences (Approval code: IR.IUMS.REC.1404.088; Approval date: April 2025). Mortality data obtained from the Ministry of Health were fully anonymized and de-identified prior to analysis; therefore, individual informed consent was waived. All procedures were performed in accordance with relevant guidelines and regulations and adhered to the principles of the Declaration of Helsinki.

### Statistical analysis

Statistical analyses were conducted to examine the characteristics of daily hospitalizations due to the selected diseases, ambient air pollutants, and meteorological variables during the period from 21 March 2019 to 19 March 2024. To assess the effect of PM_2.5_ concentrations on daily hospitalizations, generalized additive models (GAMs) based on time-series with quasi-Poisson distribution (Eqs. 1 and 2) were employed. Generalized additive models (GAMs) were selected because they are well suited for time-series analyses of air pollution and health outcomes. GAMs allow flexible modeling of non-linear relationships between mortality, long-term temporal trends, and meteorological variables through smoothing functions. Daily mortality counts often exhibit overdispersion; therefore, a quasi-Poisson distribution was used to provide robust variance estimates and reliable statistical inference. In these models, long-term temporal trends, daily mean temperature, and relative humidity were incorporated using spline smoothing functions to control for potential confounding effects. Additionally, variables with a p-value less than 0.25 were included in multivariate Poisson regression, and the hazard ratios with 95% confidence intervals were calculated for these variables.

1 $$\:Poisson\:\left({\mu\:}_{i}\right)\sim{Y}_{i}$$

2 $$\:\mathrm{log}\left({\mu\:}_{i}\right)={\beta\:}_{0}+{\beta\:}_{1}\left({PM}_{2.5}\right)+{\beta\:}_{2}\left({T}_{i}\right)+{\beta\:}_{3}\left({RH}_{i}\right)+{\beta\:}_{4}\left({P}_{i}\right)+\dots\:$$

where Y_i_ ​ is the number of observed events on day I; µ_i_ ​ is the expected mean (expected number of events); β_0_ is the intercept, and β_*n*−1_ is the regression coefficients. T_i_, RH_i_, and P_i_ represent the temperature, relative humidity, and air pressure on day i, respectively.

Since the effects of pollutants on health outcomes may be delayed, both single-day lags (lag 0–lag5) and cumulative lags (lag 01–lag 05) were examined. For example, lag 0 represents the effect of PM_2.5_ on the same day, while lag 01 indicates its combined effect on the current and previous day. The reason for choosing a lag period of 0–5 days was based on evidence reported in previous studies^22,23^ indicating that significant associations are typically observed within this time window; therefore, all analyses were conducted using lags 0 to 5. Results are reported as the percentage change, along with 95% confidence intervals (CI), in daily hospitalizations per unit increase in PM_2.5_ concentration. Model fit was assessed using the estat gof command in Stata, and multicollinearity among variables was evaluated using the variance inflation factor (VIF).

## Results and discussion

### PM_2.5_ concentration

**Fig. 2:**
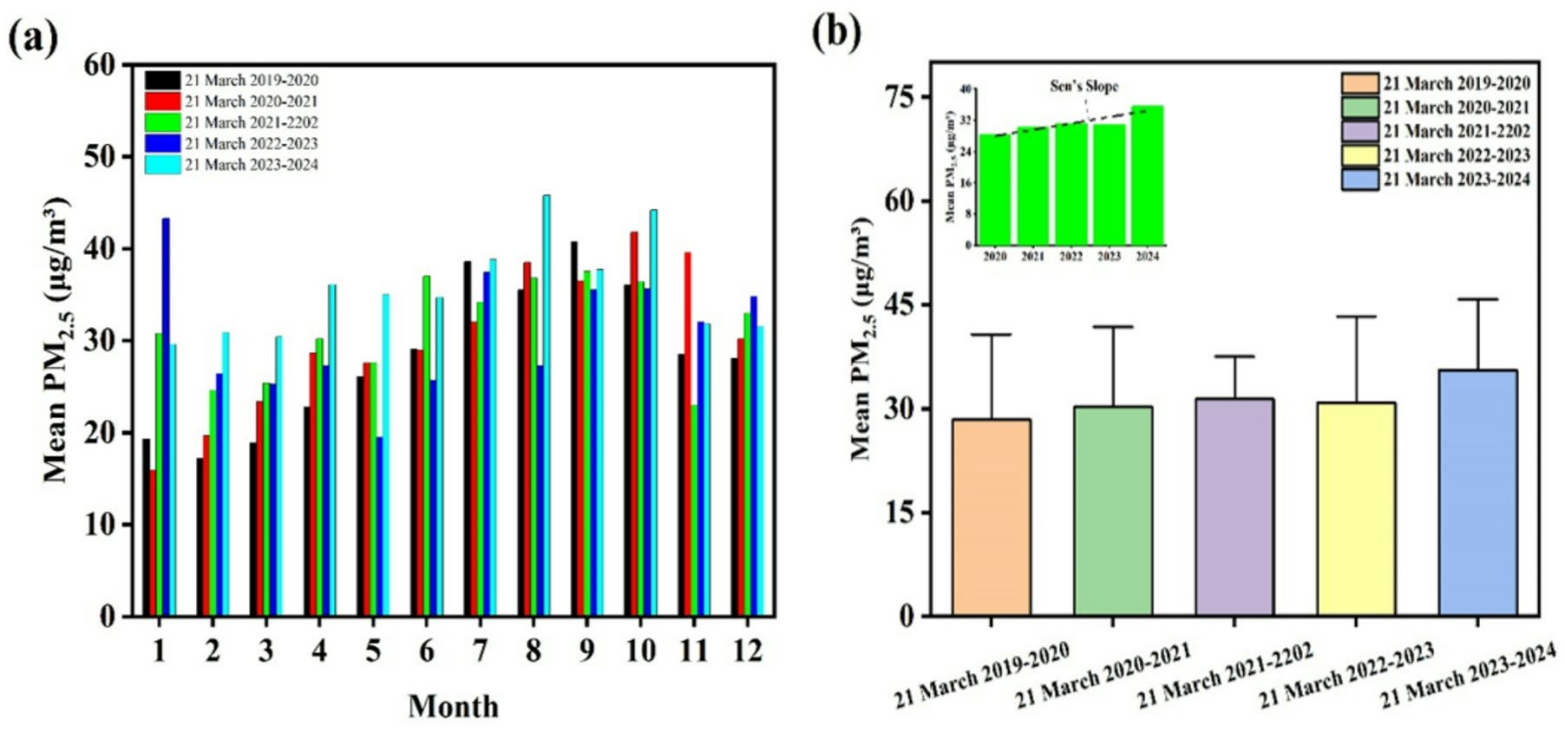
Seasonal mean concentrations of PM_2.5_ during 2019–2020, including (**a**) seasonal variations and (**b**) spatial distribution of PM_2.5_ concentrations in the study years (**b**).

Figure 2a illustrates the variations in the monthly average concentration of PM_2.5_ over a five-year period. As shown, PM_2.5_ exhibits a distinct seasonal pattern, with lower concentrations observed during the early months of the year, followed by a gradual increase from late spring to early winter. The highest levels were mainly recorded in August and September, reaching approximately 45 µg/m^3^ in 2020, which represents the peak concentration across the entire study period. An unusual peak of about 43 µg/m^3^ was also observed in March 2022, deviating from the general pattern of other years. In contrast, the years 2022–2024 exhibited milder fluctuations. Overall, in all years, the monthly mean PM_2.5_ concentrations exceeded the WHO recommended guideline of 15 µg/m³, highlighting the persistence and continuity of particulate pollution in the region. The observed seasonal variations in PM_2.5_ concentrations reflect the combined influence of meteorological conditions and pollutant emission sources on air quality. The relative reduction during the early months of the year may be attributed to more stable atmospheric conditions, increased precipitation, and enhanced natural ventilation, which facilitate pollutant dispersion. Conversely, the elevated concentrations during the mid to late months of the year are likely associated with temperature inversions, reduced rainfall, and intensified fossil fuel consumption particularly in residential heating and transportation resulting in the accumulation of fine particles in the lower atmosphere. The abnormal peak recorded in March 2022 may be linked to episodic events such as dust storms or specific climatic anomalies that occurred during that year. Furthermore, the persistent exceedance of global guideline values indicates that long-term exposure of the population to such levels of air pollution may have adverse health implications, including an increased incidence of cardiovascular and respiratory diseases. Supporting findings have been reported in previous studies. For example, Chauhan et al. measured PM_2.5_ concentrations in Varanasi from April 2019 to March 2020, reporting an annual mean of 11.34 µg/m^3^. Their results also showed higher concentrations during winter, which were attributed to meteorological conditions and elevated biomass combustion during that season^24^. Similarly, He et al. found significantly higher PM_2.5_ concentrations in winter (95.90–477.90 µg/m^3^) compared to other seasons^25^. Luong et al. reported PM_2.5_ levels ranging from 5.7 to 76.90 µg/m^3^ during 2016–2017^26^.

The analysis of PM_2.5_ concentrations over the period 2019–2024 (Fig. 2b) indicates a significant upward trend. During 2020–2021, most data values fell within the range of 20–40 µg/m^3^, with relatively higher variability. In 2021–2022 and 2022–2023, concentrations stabilized at higher levels, with a median of approximately 30 µg/m^3^, although the overall spread of values narrowed compared to the previous year. Finally, in 2023–2024, the concentrations continued to rise, with a median of around 33 µg/m^3^, representing a marked increase relative to the beginning of the study period. Statistical analysis using Sen’s Slope ( inset of Fig. 1b) confirmed these observations, showing that the mean PM_2.5_ concentration increased from 28.30 to 35.5 µg/m^3^ between 2020 and 2024. This overall pattern suggests a gradual yet persistent rise in concentrations in recent years, potentially reflecting environmental pressures, climatic changes, or other underlying contributing factors.

To better interpret these findings and clarify the association between PM_2.5_ concentrations and cardiovascular disease, the number of exceedance cases (station–day) during the period 21 March 2019 to 19 March 2024 was assessed, with results presented in Table 1. Throughout this five-year interval, the daily mean PM_2.5_ concentration in Mashhad consistently exceeded the WHO guideline value of 15 µg/m^3^; in fact, more than 90% of days during this period surpassed this threshold. By contrast, the number of days exceeding the Iranian national standard (35 µg/m^3^) was lower but showed an increasing trend, rising from 2227 days in 2019 to 3,278 days in 2024. These results clearly demonstrate that, despite the presence of national air quality standards, the urban population of Mashhad remains continuously exposed to elevated pollution levels. Moreover, the substantial gap between national limits and the more stringent global guidelines underscores the severity of the issue. The persistence of such conditions poses serious risks to public health and highlights the urgent need to reconsider air pollution control policies and strengthen national standards.

**Table 1:** Number of days exceeding WHO and National PM_2.5_ thresholds in Mashhad.

Year	WHO exceedances (PM_2.5_ > 15 µg/m^3^)	National exceedances (PM_2.5_ > 35 µg/m^3^)	Total days with data
21 March 2019–2020	7399	2227	8760
21 March 2020–2021	7637	2619	8784
21 March 2021–2022	8120	2649	8760
21 March 2022–2023	7567	2750	8760
21 March 2023–2024	7960	3278	8760

The WHO revised its PM_2.5_ guideline to 15 µg/m^3^ (24-h average), while Iran’s national standard is 35 µg/m^3^.

### Association between PM_2.5_ and ALRI mortality

**Fig. 3:**
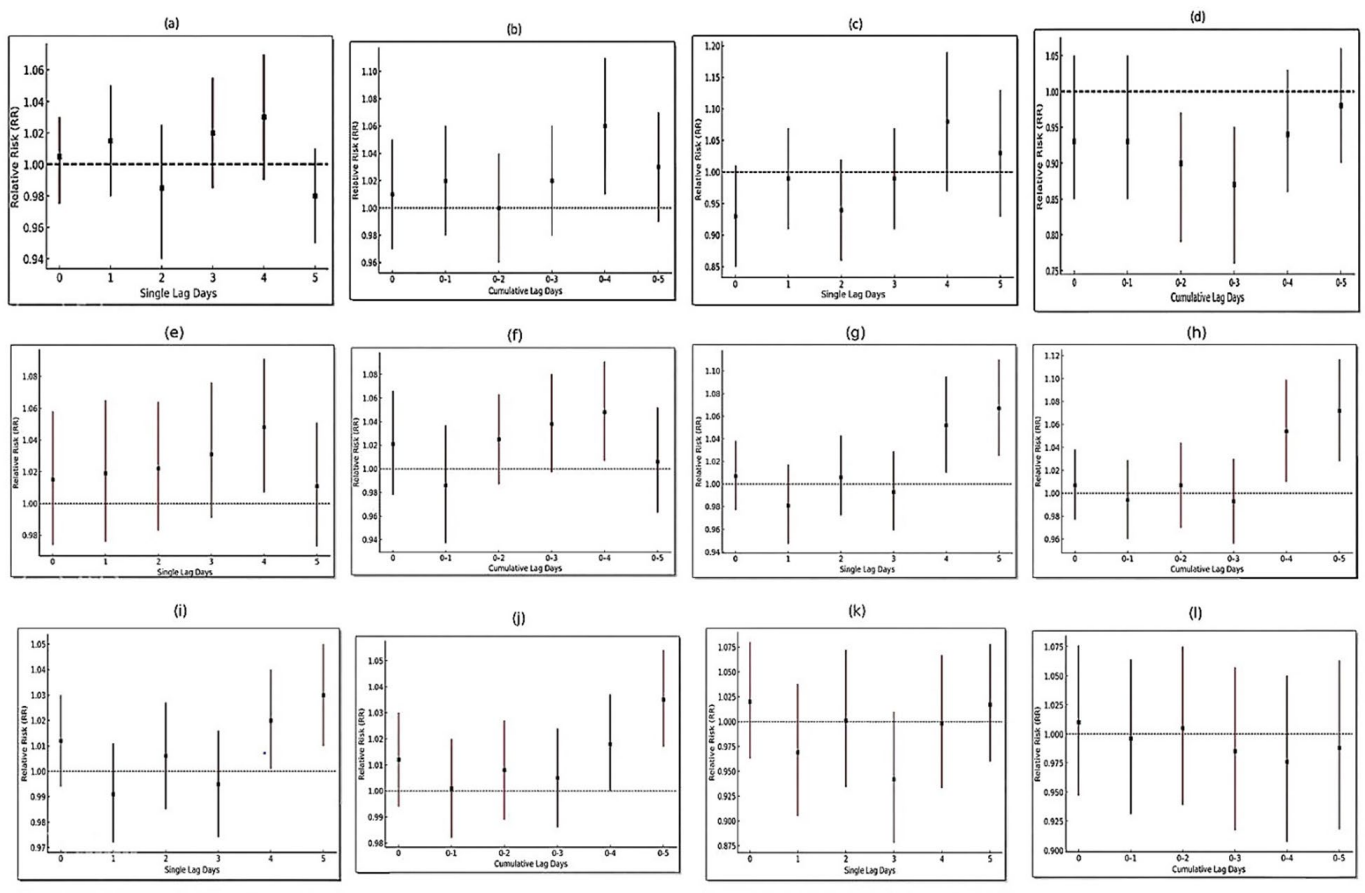
Relative risks for the association of non-cumulative and cumulative exposure to PM_2.5_ with ALRI (**a**,**b**), Asthma (**c**,**d**), COPD (**e**,**f**), stroke (**g**,**h**), IHD (**i**,**j**) and LC (**k**,**l**) mortality.

Figure 3a present the results of the non-cumulative (single-lag) effects of PM_2.5_ exposure on ALRI mortality across lag periods of 0–5 days. As shown, at Lag 0 and Lag 1, the estimated RR were slightly above 1 (1.004 and 1.014, respectively); however, these increases were not statistically significant, as the 95% confidence intervals included the null value of 1. At Lag 2, the RR declined below 1 (0.983), indicating a downward tendency, though without statistical significance. At Lag 3, the RR again rose slightly (1.020), yet remained non-significant. A notable finding emerged at Lag 4, where the RR reached 1.033 (95% CI 1.001–1.067). Since the confidence interval was entirely above 1, this result indicates a statistically significant association between 10 µg/m^3^ increase in PM_2.5_ concentration and increased ALRI mortality on the fourth day following exposure. At Lag 5, the RR decreased again below 1 (0.980), and this effect was not statistically significant.

The cumulative effects of PM_2.5_ exposure on ALRI mortality across different lag periods are presented in Fig. 3b. According to the findings, the cumulative effect peaked during Lag 0–4, with a relative risk (RR) of 1.033 (95% CI 1.001–1.067) for each 10 µg/m^3^ increase in PM_2.5_ concentrations, while changes in other lag periods were not statistically significant. A comparison of the single-lag and cumulative-lag analyses indicates that in both approaches, the impact of PM_2.5_ on ALRI mortality followed a delayed-response pattern. In the single-lag model, the strongest and only significant effect was observed at Lag 4, suggesting an increased risk of death four days after exposure to PM_2.5_. Similarly, in the cumulative-lag analysis, a significant effect was also detected during Lag 0–4, implying that the accumulated exposure over four consecutive days elevated the risk of ALRI mortality. In other words, both models consistently identified the fourth day after exposure as the critical point when the effect of PM_2.5_ became statistically significant, underscoring its important role in exacerbating ALRI-related mortality. This convergence of findings strengthens the inference that the observed association reflects a true and cumulative effect of air pollution rather than a random fluctuation. From a biological perspective, this delayed effect is plausible. Fine particulate can penetrate deep into the lungs, inducing local and systemic inflammation, oxidative stress, and impairments in mucociliary clearance. These processes, particularly in vulnerable populations such as the elderly, children, and patients with chronic conditions, can increase microbial load and weaken immune defenses, thereby facilitating the progression or recurrence of respiratory infections. Consequently, ALRI mortality is more likely to occur several days after exposure rather than on the same day or the day immediately following. Comparable evidence has been reported in previous studies. Luong et al. assessed ALRI risk among children in Ho Chi Minh City and found that with longer single-lag intervals (from Lag 0 to Lag 3), the percentage risk increased from 0.37% to 3.51%^26^. These findings emphasize that a stronger short-term response was observed in the first three days after exposure, while our findings showed the greatest effect on the fourth day (Lag 4). This delayed response may be due to regional differences in population vulnerability, pollutant composition, and access to health services between the study area and Ho Chi Minh City. Oh et al. examined the association between PM_2.5_ and ALRI hospitalizations in children, reporting that a 10 µg/m^3^ increase in PM_2.5_ at Lag 7 was associated with a 1.2% rise in ALRI admissions. The authors explained this effect through mechanisms involving oxidative stress, systemic inflammation, and impaired alveolar macrophage function, which typically develop over several days rather than immediately. Thus, the presence of multi-day lags is biologically justifiable, particularly in children, who are more vulnerable due to incomplete lung development and higher respiratory rates^27^. Unlike study of Oh et al., who observed the strongest association between PM_2.5_ exposure and ALRI hospitalizations at Lag 7 among children, our study detected the peak effect earlier, at Lag 4, in relation to ALRI mortality. This discrepancy may arise from differences in the studied outcomes (hospitalization versus mortality), population age structure, and exposure susceptibility, as children typically exhibit delayed physiological responses due to developing immune and respiratory systems. Kinzunga et al. reported that 30.72% of ALRI deaths were attributable to PM_2.5_ exposure. In their study, mortality associated with air pollution was explained through two main pathways: long-term exposure promoting chronic inflammation and oxidative stress in lung tissue, which predispose to disease development, and acute exposures acting as the final trigger for death^28^.

### Association between PM_2.5_ and asthma mortality

The findings on the single-lag and cumulative effects of PM_2.5_ exposure on asthma mortality are presented in Fig. 3c,d. According to the results, no statistically significant associations were observed during short lag periods (Lag 0 to Lag 3). Within this interval, the RR values remained close to 1, and the 95% confidence intervals consistently included the null value. This indicates that short-term exposure to PM_2.5_, whether analyzed in a single-lag or cumulative framework, did not produce a significant change in asthma mortality risk. The non-significant reductions observed at Lag 0 and Lag 2 (in both single-lag and cumulative analyses) appear to suggest a possible downward tendency rather than a definitive effect. Such patterns may reflect limitations in statistical power, natural variability in the data, or the absence of immediate impacts of PM_2.5_ exposure on asthma-related mortality.

In contrast, the results for longer lag periods indicated a positive trend approaching statistical significance. Specifically, at Lag 4 (RR 1.087; 95% CI 0.99–1.19) and Lag 0–4 (RR 1.086; 95% CI 0.99–1.19), the relative risks suggested a potential delayed or cumulative increase in asthma mortality risk by approximately 8.6–8.7% for each 10 µg/m^3^ increase in PM_2.5_ concentrations. Similarly, at Lag 5 and Lag 0–5, slight but non-significant increases were observed, further supporting the hypothesis that the effects of PM_2.5_ on asthma patients may emerge gradually over extended time windows. The convergence of single-lag and cumulative-lag findings indicates that although no definitive evidence was found for immediate effects, the observed trends highlight the importance of considering delayed and cumulative impacts. From a biological standpoint, this pattern is plausible: due to their small size and aerodynamic properties, PM_2.5_ can penetrate into the small airways, generating high oxidative stress. This oxidative stress activates inflammatory pathways, elevates pro-inflammatory cytokines and chemokines, and exacerbates allergic and asthmatic responses^29^. Similar observations have been reported in prior research. FW et al. found a significant association between asthma hospitalizations and PM_2.5_ concentrations, with an RR of 1.021 over lag days 0–5. The authors attributed this to enhanced inflammation, reduced pulmonary defense mechanisms, and exacerbation of underlying respiratory conditions caused by particulate matter^30^. In another study, Fan et al. reported a positive correlation between PM_2.5_ levels and cumulative asthma incidence with lags of 0–7 days, where each 10 µg/m^3^ increase in PM_2.5_ corresponded to a 0.49% rise in asthma incidence^31^. Horne et al. observed that the risk of asthma onset in adults peaked within the first three days of wildfire-related PM_2.5_ exposure, whereas in children, the highest risk occurred after a lag of 3–4 weeks^32^. Pini et al. reported a significant increase in hospital admissions due to asthma exacerbations at lag 0–1 and lag 0–5. Although their study differed from ours in terms of outcome (hospitalization in Pini’s study versus mortality in the present one), both investigations highlight the short-term effects of PM_2.5_ on asthma exacerbations and underscore the importance of considering lag structures and implementing public health warnings during polluted days^33^. These findings are also consistent with a study conducted in Hangzhou, China, where PM_2.5_, along with other pollutants (NO_2_ and PM_10_), was significantly associated with increased visits of asthma patients at lags 2 and 3. Despite differences in the study populations (children in the Chinese study) and outcome measures (mortality versus patient visits), both studies confirm the short-term adverse impacts of PM_2.5_ on public health^34^.

### Association between PM_2.5_ and COPD Mortality

The results of both single-lag and cumulative analyses examining the association between PM_2.5_ and COPD mortality are presented in Fig. 3e,f. In the single-lag analysis, daily exposure to PM_2.5_ was associated with an elevated RR of COPD mortality across most lag days (Lag 0 to Lag 3 and Lag 5); however, these increases were not statistically significant. The RRs in this range varied from 1.015 at Lag 0 (95% CI 0.974–1.058) to 1.031 at Lag 3 (95% CI 0.991–1.076). The only statistically significant effect was observed at Lag 4, where the RR reached 1.048 (95% CI 1.007–1.091), indicating that COPD mortality risk increased by approximately 4.8% for every 10 µg/m^3^ rise in PM_2.5_ concentration, four days after exposure. The cumulative-lag analysis revealed a similar pattern. While elevated but non-significant risks were observed at Lag 0 through Lag 0–3, a significant effect was identified at Lag 0–4, with an RR of 1.048 (95% CI 1.007–1.091), consistent with the single-lag findings. These findings indicate that cumulative exposure to PM_2.5_ over four consecutive days increases the risk of COPD mortality by approximately 4.8% for each 10 µg/m^3^ increase in PM_2.5_ concentrations. However, over Lag 0–5, the effect diminished (RR 1.006; 95% CI 0.963–1.052) and was not statistically significant. The findings of this study are consistent with existing national and international evidence^35–37^, which has documented the detrimental impacts of PM_2.5_ on both respiratory and cardiovascular diseases. In the present analysis, ALRI demonstrated a lagged effect of PM_2.5_, with a statistically significant increase in mortality observed at Lag 4. A similar pattern was also evident for COPD, indicating that fine particulate matter can exacerbate disease progression and lead to death within just a few days of exposure. In the case of asthma, although elevated risks of mortality were observed across several lags, these increases generally did not reach statistical significance. This may reflect differences in the pathophysiological mechanisms underlying asthma compared with COPD. Nevertheless, the overlap in inflammatory pathways and oxidative stress suggests that individuals with either disease are highly vulnerable to PM_2.5_ exposure^38^. Overall, the convergence of effect patterns observed for COPD and ALRI, and to some extent asthma, underscores the pivotal role of PM_2.5_ as a general risk factor for pulmonary diseases. Previous studies provide supporting evidence. For example, Li et al.^39^ reported a significant increase in hospital admissions among COPD patients with rising PM_2.5_ concentrations. Xing et al.^40^ further demonstrated that long-term exposure to PM_2.5_ and its components (including NH_4_^+^ and SO_4_^2−^) elevated the risk of COPD incidence by 10–18%, with oxidative stress, inflammatory responses, and epigenetic changes identified as potential mechanisms. Similarly, Delavar et al.^41^, through a meta-analysis, found that each 10 µg/m^3^ increase in daily PM_2.5_ concentration was associated with a 1.6% increase in COPD-related hospitalizations a figure lower than the 4.9% increase observed in our study. Wang and Liu^38^ also reported that long-term exposure to PM_2.5_ increased both prevalence and incidence of COPD, with each 10 µg/m^3^ increment raising the risk of emergency visits and hospitalizations by 1.4–2.5%. Notably, their analysis indicated that a reduction of 5 µg/m^3^ in PM_2.5_ concentration could lower COPD-related hospitalizations and mortality by up to 12%.

### Association between stroke and IHD related mortality and PM_2.5_ concentrations

The results of the short-term effects of PM_2.5_ on mortality due to stroke and IHD are presented in Fig. 3g–j. As expected, although no immediate effect was observed in the initial days of exposure, significant delayed and cumulative effects emerged. For stroke-related mortality, the RR values across Lag 0 to Lag 3 ranged from 0.981 to 1.007, none of which were statistically significant. However, a significant effect was observed at Lag 4 (RR 1.052; 95% CI 1.010–1.095), which was further strengthened at Lag 5 (RR 1.067; 95% CI 1.025–1.110). The cumulative-lag analysis showed a similar pattern, with significant associations at Lag 0–4 (RR 1.054; 95% CI 1.010–1.099) and reaching the highest level at Lag 0–5 (RR 1.072; 95% CI 1.028–1.117). These findings indicate that the cerebrovascular system is particularly sensitive to the cumulative effects of PM_2.5_, with the risk of stroke-related mortality increasing by approximately 5.2–7.2% for each 10 µg/m^3^ increase in PM_2.5_ concentrations after several days of sustained exposure.

According to the findings presented in Fig. 3i,j and a pattern similar to stroke mortality was observed for IHD-related deaths. During the initial days (Lag 0 to Lag 3), the relative risk (RR) remained close to unity and did not reach statistical significance. However, at Lag 4 a significant effect appeared (RR 1.020; 95% CI 1.001–1.040), which was further strengthened at Lag 5 (RR 1.030; 95% CI 1.010–1.050). Cumulative-lag analysis also revealed significant increases, with RR 1.018 (95% CI 1.000–1.037) at Lag 0–4 and RR 1.035 (95% CI 1.017–1.054) at Lag 0–5, representing the peak effect. Although the relative risk increases for stroke mortality (~ 7.2%) was higher than that for IHD (~ 3.5%), the substantially greater prevalence of cardiovascular disease in the general population implies that even such modest increases can contribute to a considerable disease burden. Collectively, these results suggest that the adverse effects of PM_2.5_ on cerebrovascular and cardiovascular outcomes emerge with a temporal delay and are amplified by cumulative exposure. Mechanistically, previous studies^42,43^ indicate that PM_2.5_ exposure induces systemic inflammation, oxidative stress, endothelial injury, and platelet dysfunction all of which are key pathways in the development of ischemic stroke and coronary artery disease. Furthermore, PM_2.5_ exacerbates traditional risk factors such as hypertension, insulin resistance, dyslipidemia, atrial fibrillation, and elevated homocysteine levels. Specific components of PM_2.5_, including black carbon, polycyclic aromatic hydrocarbons, and metal ions, exert direct harmful effects on the coronary and cerebral vasculature. Supporting evidence from Alexeeff et al.^44^ demonstrated in a meta-analysis that long-term PM_2.5_ exposure was associated with a 23% increase in IHD-related mortality, 24% increase in cerebrovascular mortality, 13% higher risk of stroke, and 8% higher risk of myocardial infarction. Luo et al.^45^ reported significant effects of PM_2.5_ on blood pressure: while no association F with systolic blood pressure (SBP) was observed after one month (β = 0.05; 95% CI −0.23, 0.33), prolonged exposure significantly elevated SBP (β = 1.14; 95% CI 0.54, 1.73). Ustinaviciene et al.^43^ assessed the association between stroke incidence and PM_2.5_ exposure using stratified Poisson regression from 2010 to 2022. Among middle-aged participants, 3,377 stroke cases were recorded, including 469 (13.9%) intracerebral hemorrhages and 222 (6.6%) subarachnoid hemorrhages. Similarly, Hystad et al.^46^ reported that over nearly 10 years of follow-up in more than 157,000 individuals, each 10 µg/m^3^ increase in PM_2.5_ was associated with higher risks of cardiovascular events (HR = 1.05; 95% CI 1.03–1.07), myocardial infarction (HR = 1.03; 95% CI 1.00–1.05), and stroke (HR = 1.07; 95% CI 1.04–1.10). Besides these studies, findings are consistent with those of Amsalu et al.^47^ in Beijing, although they reported the strongest effects at Lag 0–1 for cardiovascular hospitalizations. The difference in lag structures may partly reflect differences in analytical methodology: the present study employed Poisson regression with simple lag structures, while the Beijing study used a distributed nonlinear lag model with more precise adjustment for temporal trends and cumulative lags. Such methodological variation may explain why the observed effects emerged at earlier lags in Beijing but at longer delays in Mashhad. Nevertheless, the consistency of findings across distinct geographic contexts underscores the scientific importance of PM_2.5_ as a cardiovascular risk factor and highlights the urgent need for effective control policies to mitigate population exposure. In a study conducted by Khosravi et al. between 2014 and 2018 in Mashhad, a significant association was reported between PM_2.5_ exposure and both all-cause mortality and mortality due to cardiovascular diseases (RR for all-cause mortality: 1.06). In this study, NO_2_ and PM_10_ showed the strongest effects on IHD mortality. Consistent with these findings, the present study also highlights the significant role of PM_2.5_, particularly at lag days 4 and 5, in increasing mortality from cardiovascular and respiratory diseases in Mashhad. Variations in the effective lag periods may be attributed to seasonal differences, pollutant composition, or specific demographic characteristics of the study population^35^.

### Association between lung cancer (LC) mortality and PM_2.5_

Figure 3k,l present the associations between PM_2.5_ exposure and LC mortality in both single-lag and cumulative-lag models. According to the single-lag analysis, daily exposure to PM_2.5_ across all lag periods (Lag 0 to Lag 5) showed fluctuations in RR of LC mortality, but none reached statistical significance. The RR values ranged from 0.942 at Lag 3 (95% CI 0.878–1.010) to 1.020 at Lag 0 (95% CI 0.963–1.080), suggesting that the observed increases or decreases were more likely attributable to random variation in the data rather than a definitive effect of pollution.

The cumulative-lag analysis yielded similar findings: across all lag intervals (Lag 0 to Lag 0–5), the relative risks remained close to unity (e.g., Lag 0–4: RR = 0.976; 95% CI 0.907–1.050), indicating no statistically significant association between short-term PM_2.5_ exposure and LC mortality. While these short-term associations were not significant, the findings are consistent with the broader body of evidence. Unlike COPD and ALRI, where short-term and delayed effects of PM_2.5_ were evident in this study, LC is a chronic disease with a long latency period. Consequently, LC mortality is less sensitive to short-term fluctuations in air pollution, with associations primarily emerging in the context of long-term and chronic exposures. This comparison highlights that while air pollution can acutely exacerbate conditions such as COPD and ALRI, its carcinogenic effects are more strongly linked to prolonged exposure. Multiple international studies have confirmed the link between long-term PM_2.5_ exposure and LC risk. The American Cancer Society study demonstrated that each 10 µg/m^3^ increase in PM_2.5_ was associated with an 8% higher risk of LC mortality^48^. A Canadian cohort of women corroborated these results, reporting a 34% increase in LC risk per 10 µg/m^3^ increment of PM_2.5_^49^. A meta-analysis of nine European cohort studies further supported a significant association, with a hazard ratio of 1.18 (95% CI 0.96–1.46) per 5 µg/m^3^ increase^50^. In China, one study estimated that 23.9% of LC deaths were attributable to PM_2.5_ exposure^13^. Mechanistically, the association between PM_2.5_ and LC has been explained by its ability to carry toxic constituents such as sulfates, metals, polycyclic aromatic hydrocarbons (PAHs), and organic compounds. These components can initiate carcinogenesis through inflammation, oxidative stress, DNA damage, and epigenetic alterations^51^.

In contrast to our findings, which revealed no significant short-term association between PM_2.5_ exposure and LC mortality, most previous studies have reported clear evidence of a link between long-term exposure and lung cancer outcomes. For instance, the American Cancer Society cohort study and the Canadian women’s cohort both found substantial increases in LC mortality risk per 10 µg/m³ rise in PM_2.5_, suggesting that the carcinogenic impact of particulate matter is mainly driven by cumulative, chronic exposure rather than daily variations. Likewise, the ESCAPE meta-analysis across nine European cohorts confirmed a positive association between long-term PM_2.5_ exposure and lung cancer mortality. The discrepancy between our short-term null findings and these long-term associations may be attributed to the long latency period required for carcinogenesis, differences in regional pollution composition, and the relatively limited day-to-day variability in PM_2.5_ concentrations observed in our study area. Therefore, our results align with the broader understanding that PM_2.5_ acts as a chronic carcinogenic factor with effects that become evident only after prolonged exposure.

### Effects of meteorological parameters on PM_2.5_-related mortality

To evaluate the influence of meteorological parameters on mortality associated with PM_2.5_ exposure, regression analyses were conducted, and the results are presented in Table 2. The findings indicated that certain meteorological parameters were significantly associated with both ambient PM_2.5_ concentrations and disease-specific mortality outcomes. At the air pollution level, sea level pressure (SLP, *p* = 0.0003) and precipitation (PRCP, *p* = 0.0242) had the strongest significant effects on PM_2.5_ concentrations, although the explanatory power of the model was relatively low (R^2^ = 0.054). This suggests that atmospheric pressure and rainfall may contribute to short-term fluctuations in PM_2.5_ concentrations. In terms of health outcomes, the strongest association was found for ALRI mortality, where temperature (TEMP, *p* = 0.0004), SLP (*p* = 0.0001), and PRCP (*p* = 0.0055) were significant predictors, resulting in the best model fit (R^2^ = 0.277). This finding highlights the high sensitivity of ALRI mortality to meteorological conditions. Persistent effects of SLP were also observed for several chronic respiratory diseases, including asthma (*p* = 0.0017, R^2^ = 0.094), COPD (*p* = 0.0089, R^2^ = 0.138), and LC (*p* = 0.0022, R^2^ = 0.168). This pattern suggests that fluctuations in atmospheric pressure can broadly influence mortality risks from respiratory diseases. For cardiovascular diseases, precipitation was the only significant factor associated with IHD mortality (*p* = 0.0422, R^2^ = 0.133), whereas both temperature (TEMP, *p* = 0.0483) and precipitation (PRCP, *p* = 0.0149) were significantly related to stroke mortality (R^2^ = 0.121). These findings are consistent with international evidence. Ustinaviciene et al. reported that meteorological conditions such as temperature, relative humidity, and wind speed, in addition to influencing PM_2.5_ dispersion, can directly increase stroke risk. Extreme temperature events such as heat waves or cold spells have been shown to exacerbate the effects of air pollution and elevate stroke incidence^43^. Similarly, Tran et al. investigated the combined effects of temperature, relative humidity, and PM_2.5_ on COPD, demonstrating that high temperatures and very low or very high humidity, together with PM_2.5_ exposure, impaired lung function and increased COPD risk. Their study emphasized that even short-term exposure to unfavorable climatic conditions may have acute respiratory health impacts and exacerbate chronic diseases^52^. Furthermore, Fang et al. highlighted that meteorological parameters such as reduced wind speed, elevated atmospheric pressure, and a lower planetary boundary layer height (PBLH) can directly intensify PM_2.5_ pollution. These conditions hinder vertical pollutant dispersion, promote the accumulation of fine particles, and increase the likelihood of severe air pollution episodes^53^. Javorac et al. also demonstrated that atmospheric pressure, rainfall, and elevated humidity contribute to COPD exacerbations. Post-precipitation allergen release and favorable conditions for fungal growth may further heighten the risk of acute respiratory episodes, worsening diseases such as COPD and asthma^54^.

**Table 2:** Influence of meteorological parameters on PM_2.5_ concentration and disease-related mortality in Mashhad (21 March 2019 to 19 March 2024).

*P*-values	*R*-squared	Outcome
SLP (*p* = 0.0003), PRCP (*p* = 0.0242)	0.054	PM_2.5_ concentration
TEMP (*p* = 0.0004), SLP (*p* = 0.0001), PRCP (*p* = 0.0055)	0.277	ALRI mortality
SLP (*p* = 0.0017)	0.094	Asthma mortality
SLP (*p* = 0.0089)	0.138	COPD mortality
PRCP (*p* = 0.0422)	0.133	IHD mortality
TEMP (*p* = 0.0483), PRCP (*p* = 0.0149)	0.121	Stroke mortality
SLP (*p* = 0.0022)	0.168	Lung cancer mortality

## Limitations and recommendations

Although this study demonstrated significant associations between short-term exposure to PM_2.5_ and mortality from cardiovascular and respiratory diseases, several limitations should be considered when interpreting the results. First, the time-series design is restricted to short-term effects and does not allow for the assessment of long-term cumulative impacts. Second, individual-level confounding factors such as age, sex, and pre-existing conditions were not included in the model, which may have obscured some true associations. In addition, air pollution was assessed solely based on PM_2.5_ concentrations, without accounting for its chemical composition or potential interactions with other pollutants.

Furthermore, area-level socio-economic indicators were not available for the study period due to data access and privacy constraints; therefore, potential effect modification by socio-economic context could not be evaluated. Moreover, although hospital admissions may represent a sensitive indicator of short-term health effects of air pollution, consistent and harmonized hospital admission data with standardized diagnostic coding were not accessible for the entire study period. Consequently, mortality was selected as the primary outcome, as it is a robust and commonly used endpoint in time-series studies assessing short-term associations between air pollution and health outcomes.

While meteorological variables were included in the models to control for their confounding effects, a detailed characterization of meteorological patterns and the identification of region-specific PM_2.5_ thresholds were beyond the scope of this study. Defining local baseline or “normal” PM_2.5_ concentration thresholds would require long-term climatological analyses and integrated exposure assessments. Future studies could address these aspects to improve contextual interpretation of air pollution–health associations in the region.

In addition, this study did not distinguish between different sources or sequences of particulate matter exposure, such as natural dust storm events followed by anthropogenic pollution episodes. The potential combined or sequential effects of these exposure patterns on mortality were beyond the scope of the present analysis and warrant further investigation in future studies.

Future studies are recommended to apply advanced modeling approaches, such as Distributed Lag Non-linear Models or Bayesian hierarchical time-series models, to better capture complex and non-linear relationships between pollutants and health outcomes. Expanding the study period, incorporating multi-city datasets, accounting for individual-level variables, and focusing on vulnerable subpopulations would further enhance the accuracy and generalizability of the findings.

## Conclusion

Using a time-series design and quasi-Poisson regression, this study evaluated the association between daily PM_2.5_ concentrations and mortality from respiratory and cardiovascular diseases in Mashhad, Iran, during 2019–2024. Short-term effects were assessed using both single-day lags (0–5) and cumulative lags (0–5). The results indicated that PM_2.5_ exposure was significantly associated with mortality from IHD, stroke, COPD, and ALRI, while asthma and lung cancer showed near-significant trends. For all-cause mortality, the strongest lagged effects were observed at lag 4 and cumulative lag 0–4, suggesting the presence of delayed pathophysiological mechanisms. Analysis of PM_2.5_ trends between 2019 and 2024 revealed a significant upward trajectory, with daily mean concentrations consistently exceeding the WHO guideline of 15 µg/m^3^. This provides strong evidence for the detrimental effects of particulate matter on mortality due to respiratory and cardiovascular diseases. Regression analyses of meteorological parameters further indicated that ALRI had the strongest, and asthma the weakest, associations with meteorological factors. Overall, the findings confirm a significant relationship between PM_2.5_ exposure and cause-specific mortality, underscoring the urgent need for effective air pollution control strategies to mitigate the health burden.

## Data Availability

The datasets analyzed during the current study were obtained from the Iranian Ministry of Health and Medical Education and the Department of Environment. These datasets contain sensitive and confidential health and environmental information and are not publicly available due to privacy and institutional restrictions. De-identified or aggregated data may be made available from the corresponding author upon reasonable request and with permission from the data-providing authorities.
